# Bacterial microcompartments: catalysis-enhancing metabolic modules for next generation metabolic and biomedical engineering

**DOI:** 10.1186/s12915-019-0691-z

**Published:** 2019-10-10

**Authors:** Henning Kirst, Cheryl A. Kerfeld

**Affiliations:** 1 MSU-DOE Plant Research Laboratory, Michigan State University, 612 Wilson Road, East Lansing, MI 48824 USA; 2 Environmental Genomics and Systems Biology and Molecular Biophysics and Integrated Bioimaging Divisions, Lawrence Berkeley National Laboratory, 1 Cyclotron Road, Berkeley, CA 94720 USA; 3 Department of Biochemistry and Molecular Biology, Michigan State University, 603 Wilson Road, East Lansing, MI 48824 USA

## Abstract

Bacterial cells have long been thought to be simple cells with little spatial organization, but recent research has shown that they exhibit a remarkable degree of subcellular differentiation. Indeed, bacteria even have organelles such as magnetosomes for sensing magnetic fields or gas vesicles controlling cell buoyancy. A functionally diverse group of bacterial organelles are the bacterial microcompartments (BMCs) that fulfill specialized metabolic needs. Modification and reengineering of these BMCs enable innovative approaches for metabolic engineering and nanomedicine.

## What are bacterial microcompartments?

Bacterial microcompartments (BMCs) are organelles in prokaryotic cells. In contrast to those of eukaryotes, BMCs are not circumscribed by a phospholipid membrane. Instead, the barrier between the lumen of the organelle and the cytosol is formed by conserved families of proteins that assemble into a selectively permeable shell [1–5]. While the shell architecture is broadly conserved across all BMCs, the encapsulated enzymes vary widely [6]. In general, BMCs are metabolic modules, with the enzymes carrying out a sequence of biochemical reactions, and the shell serving as the interface with the cytosol.

## What defines the BMC shell?

**Fig. 1.**
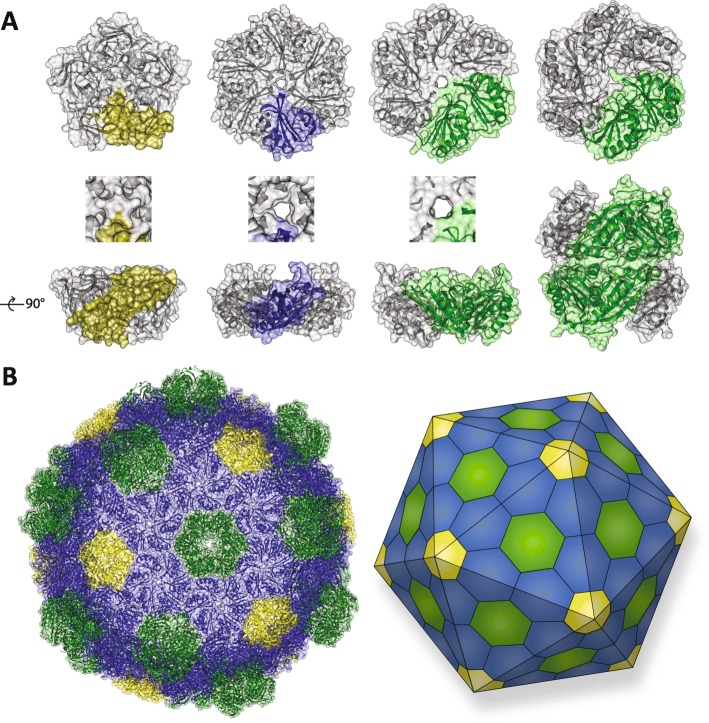
The BMC structural proteins forming a polyhedral shell. **a** The four different types of building blocks: BMC-P (pentamer monomer in *yellow*); BMC-H hexamer (monomer in *blue*); BMC-T^S^ trimer (monomer in *green*), and the double stacked BMC-T^D^ hexamer (monomer in each layer in *green*). A top view and a side view are shown, as well as a close up of the center for the BMC-P, BMC-H, and BMC-T^S^. The BMC-T^D^ pore can have an open or closed conformation (closed conformation is shown). The shell proteins have a concave and a convex side, the concave side faces outward (towards the top of the side view), while the convex side faces the BMC lumen (towards the bottom of the side view). **b** The structure of a BMC shell from *Haliangium ochraceum* and a schematic of the icosahedral symmetry (BMC-H in *blue*; BMC-T^S^ and BMC-T^D^ in *green*; BMC-P in *yellow*)

BMCs are defined by the structural proteins that compose their “membranes”. There are three structural groups of shell proteins: BMC-H (Pfam00936), which form hexagonal hexamers [1]; BMC-P (Pfam03319), which from pentagonal pentamers [7, 8], and BMC-T (a tandem fusion of the Pfam00936), which subdivide into two types: trimers (BMC-T^S^) [9–12], and stacked dimers of trimers (BMC-T^D^) (Fig. 1a) [13–15]. Pores, typically at the cyclic axis of symmetry in the hexamers, vary in size (4–7 Å in diameter) and charge, thereby contributing to selective permeability (Fig. 1a) [1, 13–19]. It has been shown that some BMC-H proteins are specifically permeable to anions like HCO_3_^−^ [20]. The BMC-T^D^ trimers can have gated pores, meaning they have an open and closed conformation (closed conformation shown in Fig. 1a) [13, 14, 18]. The stacking of BMC-T^D^ trimers creates an internal chamber with pores to the lumen of the shell and to the cytosol [9, 13, 15, 18]. Their gated pores have been proposed to operate in an airlock fashion with opening and closure controlled by ligand binding [13–15, 18, 21], apparently in a coordinated fashion across the surface of the shell [21]. These structural proteins assemble into a polyhedral shell (Fig. 1b). BMC-H proteins tile into a single layer, but some BMC-H proteins also appear to stack, similar to the BMC-T^D^ proteins [19, 22]. Recently, this has been suggested to be potentially physiologically relevant by dynamically attenuating the shell’s permeability depending on environmental conditions [22].

## How diverse are BMCs?

**Fig. 2:**
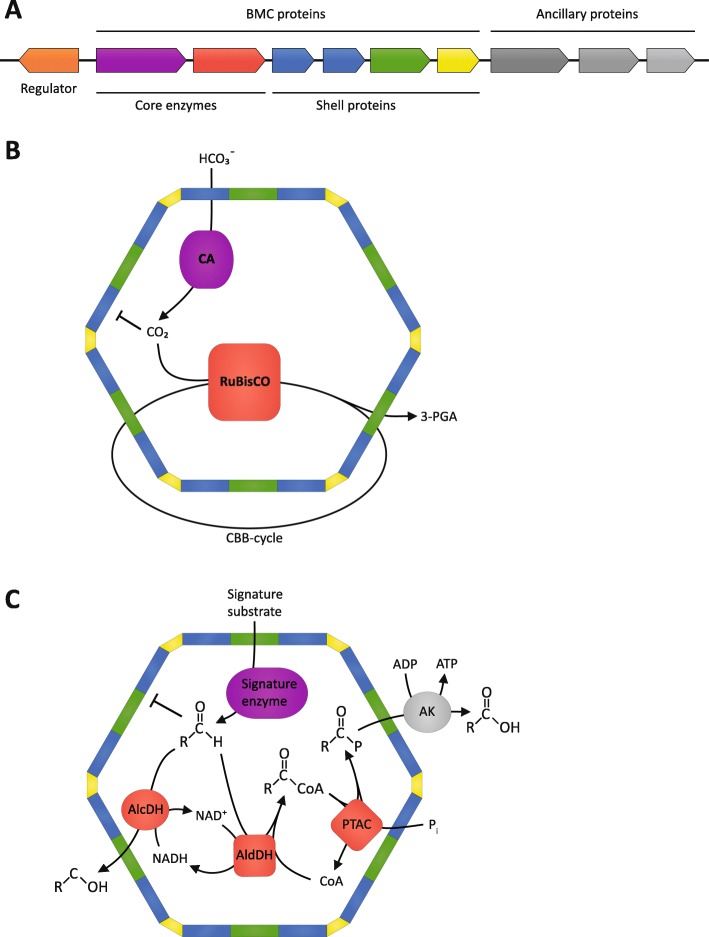
BMC genetic and metabolic modularity. **a** Schematic of a BMC gene locus containing a transcriptional regulator (*orange*) presumably controlling the expression of the BMC operon, the enzymatic core (*purple* and *red*), the structural shell proteins (*blue*, *green*, and *yellow*) forming the BMC and the ancillary proteins positioning and metabolically integrating the BMC into the cell (*gray*). **b** Schematic function of the carboxysome. The shell acts as a barrier to concentrate the CO_2_ and potentially exclude the competitive inhibitor oxygen within the BMC, enabling RuBisCO to operate more efficiently. *3-PGA* 3-phosphoglycerate. **c** Schematic function of metabolosomes. The toxic aldehyde intermediate is contained and detoxified within the BMC. *CoA* coenzyme A, *P*_i_ inorganic phosphate

BMCs are encoded in gene clusters containing the genetic information necessary to form the BMC and integrate its function with the rest of cellular metabolism [4–6, 23]. This typically includes substrate sensors (regulatory proteins), plasma membrane-associated transporters, enzymes, shell proteins, and cytoskeletal elements presumed to control positioning of the organelle (Fig. 2a). The compact arrangement of genes for organelle components and ancillary proteins that support the metabolic integration of the BMC with the rest of a cell’s metabolism likely accounts for their apparent widespread horizontal gene transfer (evident by the same type of BMC being present in very similar genetic arrangements in distantly related bacteria [4–6, 23]), illustrating the concept of “plug and play” devices in evolution.

BMC loci have been identified in 23 out of the 29 established bacterial phyla [6], and can be divided metabolically into anabolic carboxysomes and catabolic metabolosomes (recently reviewed by [5, 24, 25]). There are two distinct carboxysomes (alpha- and beta-carboxysomes), which differ in the type of RuBisCO and the conserved carbonic anhydrase they encapsulate. The carbonic anhydrase converts bicarbonate to CO_2_, the substrate for RuBisCO. The co-localization of the enzymes, and the barrier provided by the BMC shell, increases the local concentration of CO_2_, enhancing the efficiency and selectivity of RuBisCO (Fig. 2b) [26, 27].

Metabolosomes are functionally diverse. The types that have been experimentally characterized are propanediol utilizing (PDU and GRM3) [28–30], ethanolamine utilizing (EUT) [31], fucose and rhamnose utilizing (GRM5 and PVM) [32, 33], 1-amino-2-propanol utilizing (RMM) [34, 35] and choline utilizing (GRM2) [36, 37]. Even though these metabolosomes have different substrates, they share a common core biochemistry which consists of a substrate-defining signature enzyme, an aldehyde dehydrogenase (AldDH), an alcohol dehydrogenase (AlcDH), and a phosphotransacylase (PTAC) (Fig. 2c). The signature enzyme generates an aldehyde which is then processed by the AlcDH and the coenzyme A-dependent AldDH to from an alcohol and a coenzyme A derivate of a carboxylic acid. The PTAC regenerates the coenzyme A, producing a phosphate ester that is used by a kinase to generate ATP. The aldehyde intermediate is a volatile toxin, impairing protein functions and damaging DNA, which can ultimately lead to cell death [38]. The metabolosome sequesters and detoxifies aldehyde intermediates [33, 39–41] and additionally prevents carbon loss due to its volatility [42].

Our group is now updating our survey of BMC loci found in sequenced genomes. We find that the numbers and diversity of BMCs have expanded substantially since 2014 [6], due at least in part to the recent emphasis on sequencing the genomes of microbial “dark matter” [43, 44]. Some are novel BMC loci of unknown function that do not fit the metabolosome or carboxysome paradigm (unpublished data) [6], indicating that BMCs are metabolically more diverse than previously thought and function in unexplored ways to give the organism a competitive advantage.

## How do the enzymes get into the BMC lumen?

**Fig. 3.**
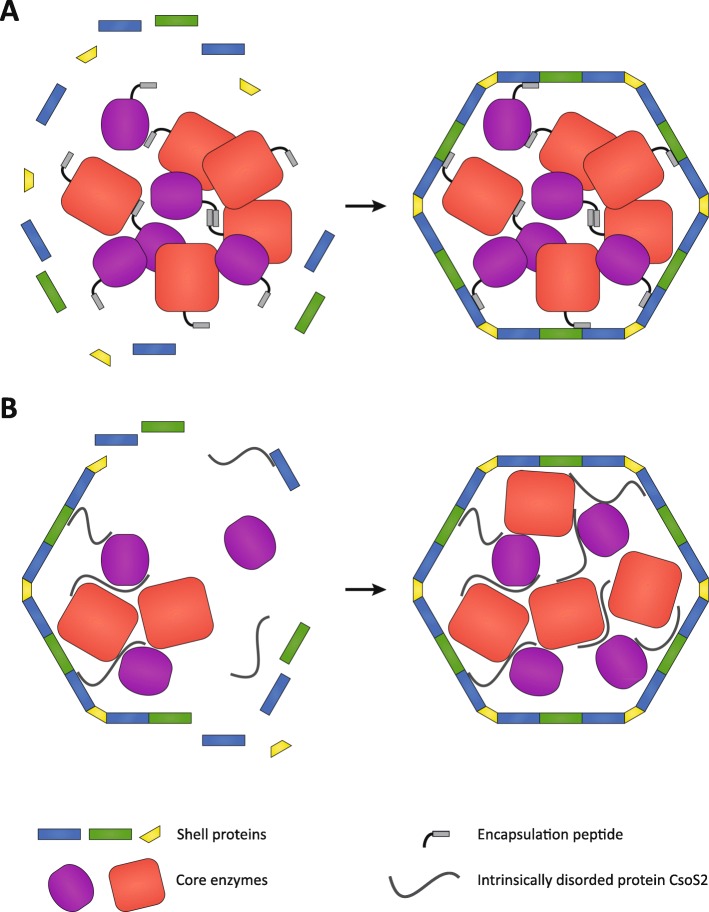
Assembly process of BMCs. **a** Core-first assembly (beta-carboxysomes and metabolosomes). Aggregation of the core enzymes is facilitated by the encapsulation peptide in combination with other assembly proteins. After aggregation the encapsulation peptide interacts with the shell proteins to from a complete BMC. **b** Concomitant assembly (alpha carboxysomes): simultaneous core aggregation and shell protein recruitment is enabled by the intrinsically disordered protein CsoS2

There is no known mechanism for proteins to cross the BMC shell. Studies of carboxysome assembly have shown that enzymatic cargo coalesces and then is encapsulated by the shell proteins (Fig. 3a) [45], or cargo and shell assemble simultaneously (Fig. 3b) [46]. Some core proteins of beta-carboxysomes and metabolosomes contain an encapsulation peptide, which are typically found at the N- or C-terminus of a cargo protein. Encapsulation peptides are composed of one or more segments of ~ 20 amino acids that are predicted to form an amphipathic α-helix [47]. These encapsulation peptides seem to facilitate aggregation of the core enzymes, and also interact with the shell proteins to form a complete assembled BMC in a core-first process (Fig. 3a) [48–53]. In the case of the alpha-carboxysomes, complete BMC formation is facilitated by an intrinsically disordered protein, CsoS2, which interacts with the cargo enzymes as well with the shell proteins [54–56]. This leads to concurrent assembly of shell and enzymatic core in alpha-carboxysomes which has been observed in detail using cryo-electron tomography (Fig. 3b) [57].

## How are BMCs being adapted for bioengineering?

The first reports of bioengineering of BMCs focused on transplanting the PDU BMC locus from *Citrobacter freundii* to *Escherichia coli* [58]. The BMC genes were expressed, enabling the transgenic *E. coli* to grow on propanediol. More recently a PDU locus was transferred to a variety of species (*E. coli*, *Salmonella bongori*, *Klebsiella pneumoniae*, *Cronobacter sakazakii*, *Serratia marcescens*, and *Pseudomonas* spp.), likewise resulting in propanediol catabolism and BMC formation [59]. In two different studies, the genes of an alpha-carboxysome have been transferred to *E. coli* and *Corynebacterium glutamicum* and their expression resulted in RuBisCO activity [60, 61].

BMC bioengineering efforts have extended to plants, with the aim to enhance CO_2_ fixation by installing carboxysomes in chloroplasts (recently reviewed by [62]). Beta-carboxysome genes [63] and an engineered alpha-carboxysome operon [64] have been transferred to chloroplasts of *Nicotiana benthamiana* and *Nicotiana tabacum*, respectively. Carboxysome-like structures formed in the chloroplasts of these transgenic plants and even allowed for photo-autotrophic growth of plant RuBisCO knockout mutants in the latter case.

Taking advantage of the self-assembly process, BMC shells can be generated by expressing the shell proteins without the cargo proteins [65–67]. Such shells can be utilized for “bottom up” approaches to construct synthetic BMCs carrying out entirely novel functions. This approach was applied to take up and store polyphosphate in *C. freundii* [68]. A polyphosphate kinase tagged with an encapsulation peptide was introduced into a PDU BMC shell. The higher amount of cellular polyphosphate in the transgenic cells was presumably caused by preventing exophosphatases access to the encapsulated polyphosphate.

## What innovative technologies are being developed to enable the full potential of bioengineering BMCs?

There are many potential applications for engineered BMCs, including serving as nano-factories for biochemical production or as novel drug delivery devices. However, methods need to be developed enabling the modification of every aspect of the shell, like loading heterologous cargo, engineering the permeability of the pores, and controlling the assembly process.

### Encapsulation of non-endogenous cargo

Being able to control which enzymes are encapsulated by the shell enables metabolically repurposing the BMC or the use of shell for entirely new applications, like bioremediation or chemical storage.

Native encapsulation peptides have been fused to heterologous cargo in order to target them into the lumen of the shells. However, this has been inefficient, with only very little cargo successfully incorporated into the BMC [66, 67, 69], indicating that we do not fully understand the determinants of enzyme encapsulation. However, engineered solutions have been developed that allow efficient and effective encapsulation of cargo. For example, the SpyTag/SpyCatcher bacterial split adhesin domains [70] have been adapted to bind cargo covalently to the inside of the shell. The SpyCatcher component was inserted into a lumen-facing loop of a BMC-T^S^ protein, and fusing the SpyTag to heterologous cargo enabled it to be specifically and efficiently encapsulated [71]. In another approach, a BMC-H protein was circularly permuted to project the N- and C-termini, which are naturally on the external side of the shell, into the lumen for fusion of cargo [72, 73]. This can be combined with using the specific interaction of two coiled-coil domains, one fused to a circularly permuted BMC-H and the other fused to the heterologous cargo to load the BMC shell [72]. Cargo can also be loaded into the shell based on electrostatic attraction. The luminal surface of a BMC-H protein was modified to be positively charged to promote the encapsulation of negatively charged biotic or abiotic cargo [74].

### Engineering shell permeability

In order to construct effective synthetic BMCs, the shell permeability would need to be tuned to fit its catalytic function. The feasibility of pore engineering has been demonstrated; changing the residues that surround the pores alters their size and permeability, without interfering with the shell assembly [17, 66, 75]. But manipulation of shell permeability is not limited to metabolite selectivity; a redox active FeS cluster has been incorporated into a BMC-T^S^ pore to enable electron flow across the shell [12]. This enables the potential for designing BMCs that require or generate electrons. A key challenge for BMC engineering is the development of tools for directly measuring shell permeability to enable rapid prototyping of shell designs.

### In vitro assembly of BMC shells

The recently developed method of assembling BMC shells in vitro will facilitate rapid prototyping [74]. Moreover, in vitro assembly allows encapsulation of toxic and/or abiotic cargo into BMCs, which is not possible in vivo; that greatly expands the versatility of BMCs to function in cell-free chemical catalysis or in nano-medicine to deliver cytotoxins to cancer cells. Furthermore, mixing functional groups carrying structural proteins in different stoichiometries allows for rapid, high-throughput screening of the most effective combination or most robust BMC shell assembly.

## What are the emerging applications of BMCs?

### Increasing efficiency in metabolic engineering

**Fig. 4.**
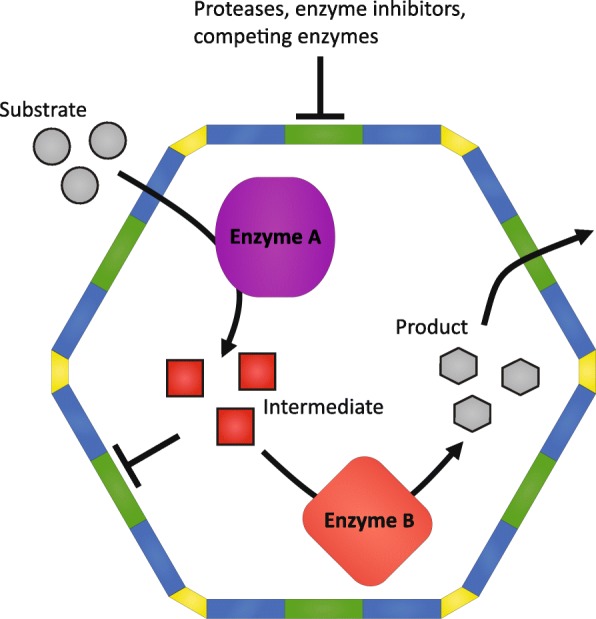
Schematic of possible synthetic BMC functions in metabolic engineering. The semipermeable shell allows a substrate to diffuse into the lumen that is then processed by enzyme A. The intermediate (*red*) can be a toxin that needs to be contained, an unstable molecule that requires fast processing, or a metabolite that can be used by off-branching metabolic pathways. In all these cases encapsulation of the enzymes into a BMC would eliminate such problems. Enzyme B can be a promiscuous or slow enzyme, operating more efficiently when given only one specific substrate in high concentrations. The shell also can act to stabilize proteins by preventing proteolytic degradation and decouples the pathway from endogenous regulation by preventing inhibitors from accessing the encapsulated enzymes

The enormous complexity of metabolic pathways, their regulation, and their crosstalk creates major obstacles for metabolic engineering, because small changes made to the system, can often have unpredictable consequences [76]. Thus, effective production strains need to go through many rounds of time-consuming optimization (recently reviewed in [77]). Ideally, an autonomous metabolic module is introduced decoupled from the cell’s regulatory and metabolic networks. Self-assembling, easy-to-modify and interspecies transferable BMCs are potential devices for the next generation of metabolic engineering (Fig. 4).

### Avoiding metabolite cross-talk

The components of the cell and its bioproducts are synthesized from a small set of precursors that feed into many different pathways [77]. If a large flux through a pathway is desired for maximum product yield, it requires a steadily available supply of precursors. Thus, metabolic cross-talk of the production pathway with existing endogenous metabolic pathways is unavoidable. Deletion of the competing pathways is not possible if these pathways are essential to the cell and downregulation needs to be fine tuned not to interfere with growth. In fact, identification of metabolic cross-talk and its effective solution is one reason for the protracted development times of efficient production strains [77]. An example of such unwanted cross-talk in metabolic engineering occurs in the synthesis of isoprenoids. Isoprenoids are a large and diverse group of natural compounds some of which are used as performance materials or as therapeutics and thus are often targeted in metabolic engineering efforts [78, 79]. All isoprenoids are synthesized by the basic building blocks isopentyl-pyrophosphate (IPP) and dimethylallyl-pyrophosphate (DMAPP). They are condensed to form the monoterpene geranyl pyrophosphate and the addition of another IPP forms the sesquiterpene farnesyl pyrophosphate (FPP). These pyrophosphate intermediates are branching off into many different isoprenoids, all of which need to be controlled for efficient production of the isoprenoid of interest. Synthetic BMCs encapsulating the production pathway can spatially insulate the intermediates from the rest of the cell, thus providing a private substrate pool for the enzyme that would otherwise need to compete for substrates with off-branching pathways if located in the cytosol.

### Improving enzyme kinetics through scaffolding and substrate concentration

Simulations indicate that compartmentalization of enzymes and the consequent local increase in intermediate substrate concentration can significantly improve catalytic turnover rates [80]. A metabolite intermediate is more likely to interact with a downstream enzyme in the compartment rather than diffusing away [81]. This concept has been used in synthetic enzyme scaffolds to increase flux through a biosynthetic pathway, recently reviewed by [82]. BMCs have naturally evolved to function as three-dimensional enzyme scaffolds and improve metabolic flux. In contrast to synthetic scaffolds developed to date, they offer a more sophisticated means of controlling metabolite flux by providing a semi-permeable shell. Because they are structurally precisely defined, loading strategies and modifications can be made accurately with a predictable outcome and thus might offer an advanced alternative to the existing synthetic enzyme scaffolds.

### Containment of cytotoxic metabolic intermediates

Bioengineering efforts are increasingly revealing issues with toxicity of intermediates due to higher steady state concentrations of metabolic intermediates when directing large amounts of carbon into the production pathway [83, 84]. One example is pyrophosphates like IPP and DMAPP needed for the generation of isoprenoids. These have been reported to be toxic to the cell when accumulated after engineering a host strain [83]. Nature’s solution to such problems is the compartmentalization of the toxic intermediate-generating metabolic step, e.g., containment of the aldehyde intermediate in metabolosomes (Fig. 3c). Next-generation metabolic engineering can take existing metabolosomes as blueprints and refunctionalize them to contain and process a specific toxic intermediate. Furthermore, they will likely prove useful for structuring metabolism in the context of cell-free metabolic engineering [85].

### Maximizing substrate specificity and minimizing metabolite damage

Like carboxysome-encapsulated RuBisCO (Fig. 3b), many enzymes are not entirely substrate specific, and damaged metabolites can be generated when an enzyme mistakenly uses a wrong substrate [86]. This is wasteful and can even be cytotoxic. The BMC shell can function to enrich the desired substrate in the vicinity of promiscuous enzymes to increase the yields of the product. This can be done by either engineering the permeability of the pores or by encapsulation of substrate-specific upstream metabolic steps.

Another form of metabolite damage is caused by spontaneous chemical reaction of the substrate with itself or with other molecules [86]. In metabolic engineering, metabolite damage remains a challenge because often little is known about spontaneous reactions of the metabolic intermediates within the chemically complex environment of the cytosol. Compartmentalizing reactions that require chemically sensitive cofactors is a natural function of metabolosomes (Fig. 3c).

### BMCs as a tool to engineer microbial communities

Many metabolosomes enable microbes to utilize specific energy, carbon, and nitrogen sources that are niche specific. Accordingly, BMCs contribute to both forming and distorting bacterial communities. An environment in which BMC-containing strains are common and potentially shape the community are hydraulically fractured shales [87, 88]. Recent studies indicate that these communities impact gas and oil production. Negative impacts arise from corrosion which is attributed to the most abundant members in these shales, *Halanaerobium* bacteria [87, 89–91]. These organisms utilize ethanolamine and the trimethylamine (TMA) produced by them is then taken up by *Methanohalophilus* bacteria [88]. The EUT (ethanolamine utilizing) BMC and many other BMC types are frequently found in the sequenced genomes of *Halanaerobium* prevailing in shales [88], suggesting that these BMCs could play a major role in shaping the subterranean bacterial community. Clearly, more research is needed to investigate the connection of the BMCs to the success of *Halanaerobium* species, which in turn might offer a potential to reshape these communities to alleviate the negative corrosiveness associated with *Halanaerobium.*

BMC-containing bacteria can also have an influence on human health by allowing a harmful species to succeed in nutrient poor environments of our body. For example, a pathogenic *E. coli* encoding a EUT BMC is able to metabolize ethanolamine and thus gains a competitive advantage over the normal intestinal flora when other nutrients are limited [92]. This can distort the bacterial community in the intestine in favor of the pathogen. A similar competitive advantage was found for the pathogen *Salmonella enterica* serovar typhimurium, also expressing a EUT BMC [93]. More recently, a choline-utilizing BMC has been characterized in uropathogenic *E. coli* [37]. Proliferation of such pathogens could potentially be prevented or treated upon introduction of a competitor to the pathogens carrying a transgenic BMC utilizing the same substrates. Such a probiotic strain could help shift the bacterial community back to a healthy intestinal flora.

## What potential do BMCs have in biomedicine?

**Fig. 5.**
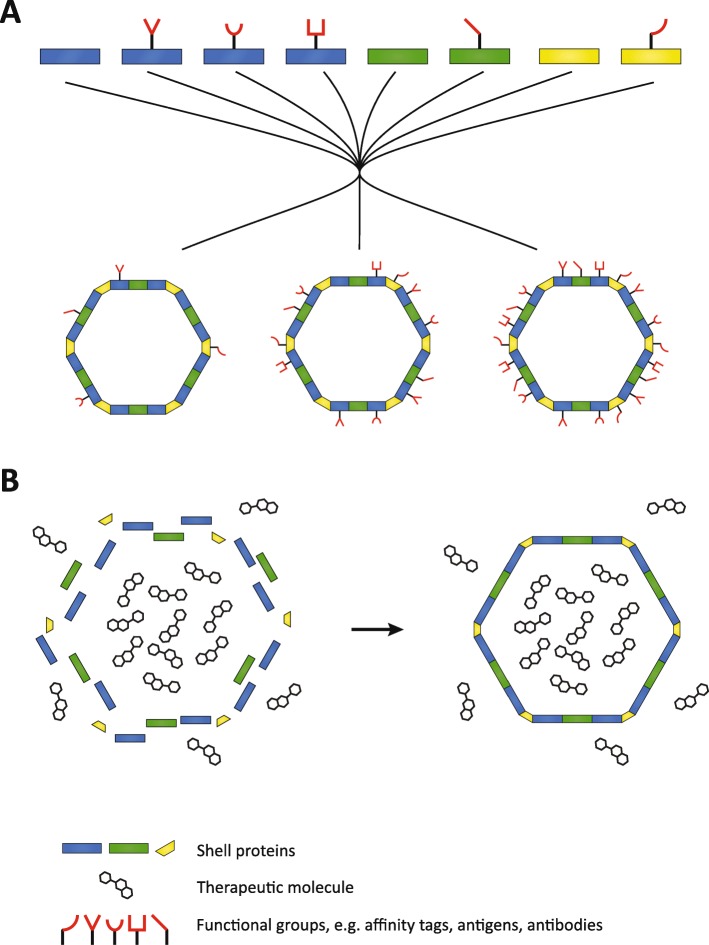
Possible applications of BMC in vitro assembly for nanomedicine. **a** High throughput testing of different combinations and densities of functional groups on a BMC. The structural proteins carrying various functional groups can be mixed together in different stoichiometries to generate many different versions of nanoparticles. **b** In vitro BMC assembly in a solution of therapeutic toxin can be used for the development of novel drug delivery systems

Nanomedicine includes the development of nanoparticles to serve as drug-delivery systems and platforms for designer vaccines [94–97]. Nanoparticles that can encapsulate therapeutic cargo have been extensively studied in recent years, yielding a diverse arsenal of useful nanostructures ranging from virus-like particles to inorganic silica nanoparticles [98]. BMC shells also have the potential to function as nanoparticle chassis for nanomedicine. For example, shell proteins could be engineered to incorporate an array of suitable peptides already developed for existing nanoparticles to facilitate active targeting of the shell to pathogens or cancer cells, tumor and cell penetration, and endosomal escape (Fig. 5a) [99]. Additionally, in vitro BMC assembly permits the encapsulation of cytotoxic therapeutics used in cancer therapy (Fig. 5b). A concern may be antigenicity of the BMC shell, limiting its application; this has yet to be tested. However, existing methods to modify the antigenicity of nanoparticles could also be used for BMCs such as the widely used PEGylation [100].

### BMCs as chassis for designer vaccines

**Fig. 6.**
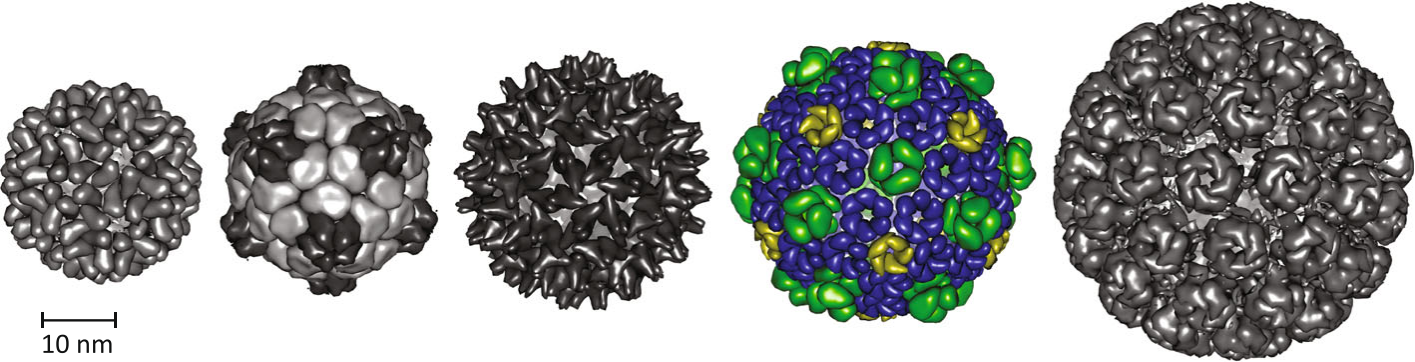
The size and geometry of virus-like particles used in biomedical engineering in comparison with the BMC shell from *Haliangium ochraceum*. From *left* to *right*: cowpea chlorotic mottle virus (PDB ID 1cwp), cowpea mosaic virus (PDB ID 5fmo), hepatitis B virus capsid (PDB ID 1QGT), *Haliangium ochraceum* BMC (PDB ID 6MZX), and murine polyomavirus (PDB ID 1sid)

Multiple parameters are important to trigger potent immune responses, including the size and geometry of the pathogen as a whole, as well as antigen density and distribution [101]. Modern vaccines can mimic these properties by utilizing nanoparticles as chassis to present antigens from a pathogen, recently reviewed by [102, 103]. BMC shells are geometrically comparable to icosahedral viruses and also have roughly the same size, ranging from 40 to 200 nm in diameter depending on the type of BMC [104, 105]. The model BMC shell from *Haliangium ochraceum* forms homogeneous particles of 40 nm diameter [104], which is very similar to virus-like particles currently used as scaffolds in biomedical engineering (Fig. 6). The *Haliangium ochraceum* BMC shell can tolerate peptide fusions to its constituent proteins, allowing for its efficient assembly [71, 74], and permitting the presentation of a diverse set of antigens. This flexibility in modifying different shell proteins in combination with the in vitro assembly method would allow high throughput sceening of different combinations and densities of antigens for the most potent immune response (Fig. 5b).
